# Accuracy of an Artificial Intelligence System for Cancer Clinical Trial Eligibility Screening: Retrospective Pilot Study

**DOI:** 10.2196/27767

**Published:** 2021-03-26

**Authors:** Tufia Haddad, Jane M Helgeson, Katharine E Pomerleau, Anita M Preininger, M Christopher Roebuck, Irene Dankwa-Mullan, Gretchen Purcell Jackson, Matthew P Goetz

**Affiliations:** 1 Mayo Clinic Rochester, MN United States; 2 IBM Watson Health Cambridge, ME United States; 3 RxEconomics LLC Hunt Valley, MD United States; 4 Vanderbilt University Medical Center Nashville, TN United States

**Keywords:** clinical trial matching, clinical decision support system, machine learning, artificial intelligence, screening, clinical trials, eligibility, breast cancer

## Abstract

**Background:**

Screening patients for eligibility for clinical trials is labor intensive. It requires abstraction of data elements from multiple components of the longitudinal health record and matching them to inclusion and exclusion criteria for each trial. Artificial intelligence (AI) systems have been developed to improve the efficiency and accuracy of this process.

**Objective:**

This study aims to evaluate the ability of an AI clinical decision support system (CDSS) to identify eligible patients for a set of clinical trials.

**Methods:**

This study included the deidentified data from a cohort of patients with breast cancer seen at the medical oncology clinic of an academic medical center between May and July 2017 and assessed patient eligibility for 4 breast cancer clinical trials. CDSS eligibility screening performance was validated against manual screening. Accuracy, sensitivity, specificity, positive predictive value, and negative predictive value for eligibility determinations were calculated. Disagreements between manual screeners and the CDSS were examined to identify sources of discrepancies. Interrater reliability between manual reviewers was analyzed using Cohen (pairwise) and Fleiss (three-way) κ, and the significance of differences was determined by Wilcoxon signed-rank test.

**Results:**

In total, 318 patients with breast cancer were included. Interrater reliability for manual screening ranged from 0.60-0.77, indicating substantial agreement. The overall accuracy of breast cancer trial eligibility determinations by the CDSS was 87.6%. CDSS sensitivity was 81.1% and specificity was 89%.

**Conclusions:**

The AI CDSS in this study demonstrated accuracy, sensitivity, and specificity of greater than 80% in determining the eligibility of patients for breast cancer clinical trials. CDSSs can accurately exclude ineligible patients for clinical trials and offer the potential to increase screening efficiency and accuracy. Additional research is needed to explore whether increased efficiency in screening and trial matching translates to improvements in trial enrollment, accruals, feasibility assessments, and cost.

## Introduction

Patients with cancer treated in multispecialty clinical settings with access to clinical trials may experience better survival and quality of life [1-5]. Cancer research involving clinical trials is essential to bring new drugs, combination therapies, devices, and procedures into clinical practice, with the ultimate goal of decreasing cancer morbidity and mortality. Implementing a program to systematically screen patients for clinical trials can improve accruals but requires dedicated and skilled staff to complete a demanding and often tedious task [6]. Identifying patients that fit complex protocol eligibility criteria is key to successful trial recruitment and enrollment [7]; however, most clinics are not optimally staffed for the time-intensive nature of manual patient screening. Emerging health information technologies leveraging artificial intelligence (AI) techniques, such as natural language processing (NLP) and machine learning (ML), can play important roles in the clinical trial-matching and enrollment processes. Matching of eligible patients to relevant trials requires retrieval of patient information buried in the electronic health record (EHR) and extensive knowledge of complex exclusion and inclusion criteria for each trial protocol. Therefore, an automated technology that enhances efficiencies of eligibility screening for diverse cohorts of patients and large portfolios of clinical trials holds great promise for advancing cancer translational and research activities.

In oncology practices, clinical decision support systems (CDSSs) designed for cancer clinical trial matching have the potential to assist research program managers, trial coordinators, principal investigators, and cancer care providers with the eligibility screening process [8]. This assistance is needed, as the time and effort required to identify trials for individual patients increases the burden on already overstretched research and clinical care teams, but also poses a potential barrier to trials being offered to eligible patients with cancer. Protocols often include numerous complex inclusion and exclusion criteria that must be evaluated for each patient, and depending on the number of active clinical trials, research teams may need to screen and evaluate patients against a long list of possible trials. Automation of the screening process with trial-matching tools can reduce screening time and research team fatigue, thereby increasing coordinator availability to address other patient and provider barriers to clinical trial enrollment [9-12].

Sponsors of clinical trials typically seek to open new clinical trials at sites based on the expertise of the principal investigator, his or her track record of trial enrollments, as well as results of site feasibility questionnaires that may or may not accurately reflect the potential for enrollment of patients who meet eligibility criteria for the trial. An automated trial-matching system can help identify factors associated with accrual rates, including common reasons for patient exclusion, and inform discussions regarding eligibility criteria.

Watson for Clinical Trial Matching is a CDSS designed to interpret clinical trial protocols written in natural language and patient information from EHRs and provide just-in-time information to determine patient eligibility for clinical trials. The CDSS integration with an EHR facilitates intake of structured data (eg, laboratory values, demographics) and processing of unstructured information (eg, pathology reports, clinical notes) with NLP. This enables an assessment of patient eligibility across studies that have been ingested into its trial corpus (ie, ClinicalTrials.gov trials, sponsor- or investigator-initiated trials).

There are two types of approaches for the CDSS to screen and match patients to clinical trials. The first approach involves identifying the cohort of potentially eligible patients for a trial, referred to as trial-centered matching; specifically, for an individual trial, which patients among a cohort match to the trial inclusion and exclusion criteria. Trial-centered analysis by the CDSS can provide feedback to a study team on trial feasibility, including recognition of criteria that commonly lead to patient exclusion. This information can help estimate the projected site enrollment or generate protocol modifications to eligibility to optimize patient inclusion. The second approach involves identifying appropriate clinical trials for a patient, or patient-centered matching; specifically, for an individual patient, which trials among a portfolio of options match to the patient and his/her tumor characteristics. Patient-centered analysis by the CDSS can provide a ranked list of trials to clinicians or research teams at point-of-care or be used for just-in-time screening when patients contact cancer centers with interest for clinical trial opportunities.

The CDSS used in this study was initially designed to support patient-centered matching. In the current study, however, we report the evaluation of a trial-centered matching approach by the CDSS to identify eligible patients for each of 4 different clinical trials from a pool of patients with breast cancer treated at Mayo Clinic (Rochester, MN), a National Cancer Institute–designated comprehensive cancer center. The purpose of this pilot study was to determine the accuracy, efficiency, feasibility, clinical validity, and performance of the CDSS using a trial-centered matching approach.

## Methods

### Institutional Review Board Review

This study was conducted under an exemption from the Western Institutional Review Board (WIRB) as a technology pilot for epidemiologic research (Protocol 20152322). The WIRB determined that this research met requirements for waiver of consent. This pilot study was also approved by the Mayo Clinic Institutional Review Board. This pilot was not intended to direct patient care or recruitment of patients into trials. All evaluations on patient data were performed in a retrospective manner. For actual trial participation, patients were evaluated via the standard manual screening process at Mayo Clinic.

### CDSS Description and Training

This study evaluated a trial-centered approach by the Watson for Clinical Trial Matching CDSS system in a research setting. The core NLP and ML technologies designed for patient-centered matching within the system have been described elsewhere and will not be detailed in this manuscript focused on performance evaluation [13-15]. The CDSS uses NLP to determine cancer-specific attribute values from structured and unstructured deidentified data sources. In developing the CDSS, specific attributes for cancers, such as cancer stage, cancer subtype, genetic markers, prior cancer therapy, surgical status, and pathology, as well as attributes needed for trial consideration, such as therapy-related characteristics, were defined by subject matter experts (SMEs), including clinical specialists and PhD-level nurses. The NLP was trained through iterative teaching cycles with clinical information obtained from patient cases [16,17]. During these training cycles, SMEs reviewed partially trained outputs from the CDSS to identify initial attributes and values, as well as correct system outputs by providing supporting information and evidence as needed. Corrected outputs were given to developers for additional system training. This process was used to iteratively create a ground truth and allowed for greater scalability and agility during the system development process. Medical logic algorithms allowed the CDSS to prioritize clinical information when determining attribute values when two values for the same attribute were available (eg, mastectomy was prioritized over lumpectomy) [18,19]. The CDSS’s learning was continual as patient cases were processed and as medical knowledge advanced.

For the NLP process of trial ingestion, the CDSS used protocol inclusion and exclusion criteria from analysis of several thousand trials available from ClinicalTrials.gov. In this study, the Novartis protocol library was made available to Mayo Clinic, and the full inclusion and exclusion criteria from 4 breast cancer trials (ie, NCT02069093, NCT01633060, NT02437318, and NCT01923168) were ingested into the CDSS. NLP training at the protocol level was conducted by processing PDFs of the final readable trial protocols, including amendments approved by the WIRB. The CDSS applied NLP against the full protocol criteria and an evaluation file was provided to Novartis for protocol disambiguation. The disambiguation file indicated which criteria required clarification; clarification was provided with input from Novartis. Therefore, trial ingestion errors were corrected and not evaluated as part of this study.

### Study Population and Analytic Methods

Based on binomial distribution to detect accuracy of at least 80% with a power of 90% and a probability of error at the α=.05 level, a minimum sample of 172 individuals was required. We identified patient records suitable for inclusion in this retrospective pilot study from a population of patients with breast cancer treated in the medical oncology clinic at Mayo Clinic in Rochester, Minnesota, between May and June of 2017 with at least one unstructured health record note for processing by the CDSS.

The CDSS processes structured and unstructured patient data contained in the EHR (including medical oncology progress notes, pathology records, surgical reports, and laboratory values) to derive patient- and tumor-specific attributes. In this study, two groups of patient records were evaluated. Group 1 was comprised of a subset of patients that had been previously processed by the CDSS with a patient-centered approach, during which time any missing or conflicting attributes were resolved through human intervention. Group 2 patient records were processed solely by CDSS without additional human verification; any missing or conflicting attributes were marked as “unknown” by the system.

### Gold Standard

To establish a gold standard for eligibility determinations, attribute filters for the 4 preselected clinical trials were established according to tumor stage (metastatic or nonmetastatic breast cancer), patient setting (neoadjuvant/preoperative or adjuvant/postoperative setting), tumor HER2 status (positive or negative), and tumor hormone receptor status (positive or negative). One or more qualified staff (nurse abstractors) then manually reviewed patient EHRs, screened trial eligibility based on the attribute filters, and made determinations to “include” or “exclude” patients if their data attributes matched those of each individual trial.

To measure the reproducibility of manual review, a random subset of 38 breast cancer cases from Group 2 (those whose attributes were determined solely by the CDSS) were rereviewed by two additional reviewers, and interrater reliability between these additional reviewers and the gold standard manual review was calculated. The additional reviews were performed by trained breast oncology clinical research coordinators from the Mayo Clinic. For all 38 cases, the two additional reviewers repeated the same sequence of steps performed by the initial reviewer, using the same set of relevant data from filter parameters described above. Interrater reliability was assessed using Cohen κ for each of the additional reviewers compared to manual review (ie, two pairs). A single Fleiss κ coefficient was also calculated for three raters (ie, the two additional reviewers and the gold standard manual). Significance of differences was analyzed using the Wilcoxon signed-rank test.

### CDSS Performance Evaluation

The CDSS abstracted the same attributes from the EHR and determined patient eligibility by matching them with those attribute filters assigned to each trial, including tumor stage, patient setting, and tumor HER2 and hormone receptor status. The CDSS eligibility determinations were next compared to manual classifications using confusion matrices constructed by cross tabulation of inclusion and exclusion determinations from the CDSS versus manual reviewers.

The CDSS clinical performance was assessed for its predictive accuracy, sensitivity, specificity, positive predictive value, and negative predictive value using manual review as the gold standard. Discrepancies in clinical trial matches (inclusion/exclusion determinations) between the CDSS and the manual review were identified and evaluated by independent SMEs with breast cancer clinical expertise and knowledge of the CDSS’s trial-matching processes. All discrepant determinations were resolved by SMEs, categorized by type, and recorded. Types of discrepancies include the following: manual screening errors (human error), incorrectly derived by the CDSS (machine error), unsupported CDSS functionality (CDSS untrained for all breast cancer clinical scenarios, such as multiple primary tumors), filter parameters (differences in patient setting or tumor attribute without error, such as when the CDSS system’s medical logic did not include all variations of reasoning used in practice for estrogen receptor/progesterone receptor interpretation), limited records provided (insufficient data available), and project design errors (due to patient tumor attributes or setting changing over the time frame for which the patient EHR was used for the study).

## Results

### Study Population

The study sample included 327 patients with breast cancer. From the original sample, 4 patients were removed due to an unsupported disease type (noninvasive breast cancer) and 5 patients were removed as duplicates, resulting in a total of 318 patients with breast cancer.

### Reproducibility of Manual Screening

Manual review of breast cancer cases was employed to create the gold standard for this evaluation. Interrater reliability for manual assignment was substantial as Cohen κ between the gold standard and each of the two additional reviewers was 0.60 and 0.77, respectively. Fleiss κ coefficient across all three reviewers was 0.64. No statistically significant differences in assignment were detected (*P*=.16).

### CDSS Accuracy in Eligibility Determination

#### Group 1

Group 1, with attributes verified by humans as described in the Methods section, included 117 breast cancer cases. The CDSS accuracy of trial eligibility determinations for Group 1 (included/excluded) was 90.6% overall. Sensitivity (true positive rate) was 82.1%, and specificity (true negative rate) was 93.3%. The mean accuracy for this group was determined for the following filters: metastatic stage (95.4%), neoadjuvant setting (100%), HER2 status (88.9%), and hormone receptor status (93.5%; all results shown in Table 1). Discrepancies (Table 2) included 5 false positive values (originating from 4 filter parameter errors and 1 manual screening error) and 6 false negative values (3 from filter parameters, 1 due to manual screening error, 1 incorrectly derived by the CDSS, and 1 error from an unsupported CDSS functionality).

**Table 1:** Clinical decision support system accuracy by cohort.

Cohort	Overall eligibility (%)	Metastatic breast cancer (%)	Neoadjuvant setting (%)	HER2 status (%)	Hormone receptor status (%)
Group 1	90.6	95.4	100	88.9	93.5
Group 2	87.6	90.4	87.2	93	88.6

**Table 2:** False positive, false negative, and discrepancy type by cohort.

Cohort and discrepancy type(s)		Counts
**Group 1, false positive (n=5)**		
	Project design errors	4
	Manual screening errors	1
**Group 1, false negative (n=6)**		
	Filter parameters	3
	Manual screening errors	1
	Incorrectly derived by the CDSS^a^	1
	Unsupported CDSS functionality	1
**Group 2, false positive (n=7)**		
	Manual screening errors	5
	Incorrectly derived by the CDSS	2
**Group 2, false negative (n=18)**		
	Incorrectly derived by the CDSS	9
	Limited records provided	3
	Unsupported CDSS functionality	3
	Manual screening errors	2
	Filter parameters	1

^a^CDSS: clinical decision support system.

#### Group 2

In Group 2, a total of 201 cases were processed without human reconciliation of attributes. Inclusion/exclusion determinations were based solely on attribute abstraction and trial matching by the CDSS. The mean system accuracy of trial eligibility determinations of Group 2 (included/excluded) was 87.6%. Sensitivity (true positive rate) was 81.1%, and specificity (true negative rate) was 89%. The mean accuracy was determined for the following filters: metastatic stage (90.4%), neoadjuvant setting (87.2%), HER2 status (93%), and hormone receptor status (88.6%; all results shown in Table 1). Since the system processed information without human verification of attributes, unresolved attribute conflicts that led to discrepancies in trial inclusion/exclusion determinations were classified as filter parameter errors. Discrepancies (Table 2) included 7 false positive values (originating from 5 manual screening errors and 2 incorrectly derived by the CDSS) and 18 false negative values (9 values incorrectly derived by the CDSS, 3 with limited records provided, 3 related to an unsupported system functionality, 2 manual screening errors, and 1 value related to filter parameters).

## Discussion

### Principal Results

Screening for clinical trials is a complex and laborious process. This study demonstrated that an AI CDSS can automate eligibility screening accurately and identify potentially eligible patients with breast cancer with a wide variety of clinical characteristics for clinical trials. The clinical trial eligibility screening tool had a mean accuracy of 90.6% after attribute validation by research staff, which is part of the normal clinical workflow when this CDSS is used as a patient-centered solution in the practice. CDSSs such as the one used in this study can aid humans in the process of finding clinical trial matches for patients and replace the slower manual process of screening by search of EHR and eligibility criteria for each of many trial protocols with automation. The system facilitates and engages clinical staff in the completion of tasks that require human intervention, such as attribute verification and resolution of conflicting attributes. Such conflicts can arise from abstraction of attribute values from different sources within the EHR that lack consistency. This CDSS was not intended to make eligibility determinations without human interaction, but it nonetheless exhibited an accuracy of 87.6% without attribute validation by humans.

Interrater reliability of manual eligibility determination demonstrated substantial but not perfect agreement, illustrating a gap that might be filled by a combination of human and machine. Some of the manual screening errors identified in this study included marking a tumor HER2 status as unknown when the information was available in the EHR, recording incorrect hormone receptor status (estrogen receptor and/or progesterone receptor values), or failing to include attributes that changed with subsequent testing. These errors would most likely have been corrected by the combination of CDSS attribute ingestion and human verification. Errors in attribute abstraction by the system included labeling a patient as metastatic based only on disease in regional lymph nodes or annotating T3 as bone metastases when T3 referred to another clinical test or reference. In a few cases, the actual content of the unstructured notes was insufficient for the system to determine trial eligibility. In addition, the system’s medical logic did not include all variations of reasoning used in practice. For example, weakly positive hormone receptor values scored as positive may be interpreted as negative in clinical practice based on the tumor biology or behavior.

Several sources of discrepancies in trial eligibility determinations were artifacts of the study design that are unlikely to be seen in practice. For example, manual reviewers were instructed to rely solely on information explicitly documented in the medical record, without data that might be obtained from clinical inference. For patients in Group 1 with human verification of attributes, the CDSS required the end user to select a correct value when two different values for the same attribute were found within the same source document with the same date. Any attributes that might have conflicting values in Group 2 (lacking human verification) were marked as unknown by the CDSS. Overall accuracy of the system as typically used in the clinic would be expected to be closer to that obtained for Group 1 than Group 2, as verification by humans is recommended for use of the CDSS in practice.

### Limitations

There were several limitations of this study. First, the study included a relatively small number of patients with cancer from a single academic medical center and a small number of trials for breast cancer. The findings may not generalize to other settings or cancer types. Patients with multiple primary cancers, including patients with bilateral breast cancer, were not supported by the CDSS at the time of the study. Conflicting values in the CDSS were not used by the system to determine eligibility, although all data were available to manual reviewers in the EHR. The relatively fewer patients in the cohort processed by the system in Group 1 (with human verification) lacked the statistical power of the cohort of patients in Group 2 (without human verification).

### Future Work

Research is underway to evaluate system performance related to other cancer types, and this is anticipated to be successful given patient-centered matching across multiple cancers has been demonstrated [14,15]. Additionally, research to evaluate trial-centered matching scalability toward a larger volume of trials is in progress. There are also opportunities to expand patient and tumor attribute training to reflect other common and more nuanced eligibility criteria, such as prior therapies and medical comorbidities. Additional studies will be necessary to evaluate the effectiveness in translating enhanced screening into increased enrollment in clinical trials. Although this work provides evidence of the ability of technologies to expedite the trial-matching process, and automation of this process can facilitate unbiased patient screening for clinical trials, multifactorial barriers to trial recruitment remain, including racial and ethnic disparities. Further innovation and research are needed to identify strategies to address such inequities.

### Conclusions

In this study, we demonstrated the ability of an AI CDSS to screen a cohort of patients with breast cancer and determine eligibility for 4 clinical trials with very good accuracy. AI-based CDSSs have the potential to optimize the efficiency and accuracy of the trial-matching process, with the overall goal of increasing clinical trial enrollment and completion of trial objectives. This may ultimately expedite the approval of lifesaving drugs to improve cancer outcomes.

## Abbreviations

AI: artificial intelligence
CDSS: clinical decision support system
EHR: electronic health record
ML: machine learning
NLP: natural language processing
SME: subject matter expert
